# Nationwide study of respiratory-related hospitalisations and deaths in preterm children in Brazil: a registry-based study

**DOI:** 10.1186/s12931-025-03449-6

**Published:** 2025-12-13

**Authors:** Thiago Cerqueira-Silva, Pilar T. V. Florentino, Aline dos Santos Rocha, Rita de Cássia Ribeiro Silva, Maurício L. Barreto, Enny S. Paixão

**Affiliations:** 1 Faculty of Epidemiology and Population Health, London School of Hygiene & Tropical Medicine, Keppel St, London, WC1E 7HT UK; 2 Laboratório de Medicina E Saúde Pública de Precisão, Fundação Oswaldo Cruz, Salvador, Brazil; 3 Centro de Integração de Dados E Conhecimento Para a Saúde (CIDACS), Fundação Oswaldo Cruz, Salvador, Bahia Brazil; 4 School of Nutrition, Federal University of Bahia (UFBA), Salvador, Brazil

## Abstract

**Background:**

Preterm birth and respiratory diseases disproportionately affect low-and middle-income countries. Although preterm birth is a major contributor to the burden of respiratory morbimortality in early childhood, most evidence comes from high-income settings. To address this gap, we examined respiratory-related hospitalisations and deaths among preterm children in Brazil.

**Methods:**

We conducted a population-based cohort study using the CIDACS Birth Cohort, which included all live births in Brazil from January 1, 2011, to November 30, 2018. Preterm infants were defined as infants born before 37 weeks of gestation. We examined respiratory-related hospital admissions and deaths in children under five. Mean ratios (MR) and 95% confidence intervals (CI) were estimated using the Ghosh-Lin model; hazard ratios (HR) were estimated using Cox models. Maternal characteristics were adjusted through inverse probability weighting, with treatment probabilities estimated via entropy balancing.

**Results:**

The study included 3,239,563 live births, with 288,466 (8.9%) classified as preterm. The MR for under-five respiratory hospitalisation, comparing preterm to term births, was 1.40 (95%CI:1.38–1.42), peaking at 1.68 (1.63–1.72) between 28 and 90 days, declining to approximately 1.18 (1.10–1.28) at the fourth year. For respiratory disease deaths, the under-five HR was 3.94 (3.62–4.30). Respiratory-related mortality was highest between 28–90 days of age, with an HR of 4.66 (4.00–5.43), decreasing to 1.25 (0.62–2.51) by three years of age.

**Conclusion:**

Preterm newborns have a higher risk of respiratory illness than full-term children, particularly in their first year. This understanding can guide health strategies to address premature birth issues by identifying important periods of vulnerability.

**Supplementary Information:**

The online version contains supplementary material available at 10.1186/s12931-025-03449-6.

## Introduction

Preterm birth, defined as delivery before 37 weeks of gestation, is associated with a range of short- and long-term adverse outcomes, including physical and neurodevelopmental impairments, as well as an increased risk of mortality [1–3]. Globally, the prevalence of preterm birth varies from 4 to 16% [4], whereas in Brazil, it is 11% [5]. In 2019, complications related to preterm birth accounted for approximately 900,000 deaths [4, 6], making prematurity the leading cause of death among children under 5 years old [7].

Preterm infants face underdeveloped lungs and immature immune systems, exacerbating their vulnerability to lung-related disorders, respiratory infections, and hospital admissions due to respiratory complications [8, 9]. While this risk decreases with increasing gestational age [9], even late preterm infants (34–36 weeks) are significantly more likely to develop respiratory distress syndrome and other respiratory conditions compared to term infants [10, 11]. Despite the highest burden of preterm births occurring in low- and middle-income countries (LMICs) [12], most studies investigating these risks have been conducted in high-income countries such as Canada [11], the United States [2], and the United Kingdom [9]. Some small studies conducted in LMICs have shown an increased risk of lower respiratory tract infections and asthma in preterm infants [13–15]. However, the broader adverse respiratory effects of preterm birth in these settings remain unexplored. This results in a significant gap in data from LMICs, where the burden of preterm birth is often higher due to limited healthcare resources and high rates of infectious diseases [10, 12].

Furthermore, prior research often suffers from methodological limitations, such as focusing solely on the first hospitalisation and failing to account for multiple hospitalisations during the study period [2, 11, 16, 17]. Additionally, many studies do not adjust for the higher mortality rates in preterm children compared to term children, leading to an underestimation of the true burden of respiratory complications in this population [16–18].

The present study aims to provide information about the burden of respiratory morbidity associated with preterm birth in children under five years of age in an LMIC. We used linked data from the poorest half of Brazilians applying for social programmes in the country [19] to evaluate the incidence of respiratory-related hospitalisations and deaths among children under five years of age. We also employed a robust approach that accounts for recurrent events and the increased risk of death in preterm children, ensuring a more accurate estimation of the true burden of respiratory complications in this population than in previous studies.

## Methods

### Study design

We conducted a cohort study using nationwide data from live births in Brazil (CIDACS Birth Cohort) from 1 st January 2011 to 30th November 2018. This cohort links information from the (i) Live Birth Information System (*Sistema de Informação de Nascidos Vivos*, SINASC), which records gestational age, birth weight, and maternal demographics; (ii) the Unified Registry for Social Programmes (*Cadastro Único*, CadUnico), which contains socio-economic data for families enrolled in Brazil’s social programmes; (iii) the Mortality Information System (*Sistema de Informação em Mortalidade*, SIM), which records the date and cause of death; and the (iv) Hospitalisation System (*Sistema de Internação Hospitalar—Sistema Único de Saúde*, SIHSUS), which notes the date and cause of hospitalisation. The cohort's baseline is the CadUnico database, reflecting the poorest segment of the Brazilian population [19].

The validation process of the linkage was done by clerical review of 2000 matches; the mean sensitivity and specificity linking SINASC and CadUnico were 95%, the values for linking SINASC and SIM were 93%, and SINASC and SIHSUS were 99%. Details about the linkage algorithm and quality of the databases were previously published [20].

### Participants

We excluded: (i) Births with missing data on gestational age; (ii) births with estimated gestational age using last menstrual period or missing information; (iii) Births that took place before 22 weeks or after 45 weeks of gestation or with a birthweight below 500 g or higher than 6000 g; (iv) Women who delivered multiple live births; (v) Live births without a documented place of residence; (vi) Data inconsistencies, such as conception dates of consecutive births occurring less than 220 days apart or records with different birthdates of the children across databases; (vii) women aged less than 10 or higher than 49 years.

The exclusion of live births with estimated gestational age using the last menstrual period was intended to prevent the risk of misclassifying term births as preterm, as previous work has shown a skewed distribution in birth weights among preterm babies based on gestational age from LMP [21]. We provide details on the distribution of size for gestational age by method in the supplementary Table 1.

### Exposure and covariates

The main exposure was preterm delivery, categorised as preterm (< 37 weeks) and term (≥ 37 weeks). The following categories for preterm were analysed in subgroup analysis: < 28 weeks (extremely preterm), 28–31 weeks (very preterm), 32–36 weeks (moderate to late preterm). The preterm status was measured using completed gestational week at birth as recorded in the SINASC dataset.

The covariates are maternal age at birth, marital status, parity, race/ethnicity, year of pregnancy, previous fetal loss, adequacy of number of prenatal appointments, years of schooling, state of residency, and municipality deprivation level (Brazilian deprivation index). The adequacy of the number of prenatal appointments was calculated according to the recommendations of the Brazilian Ministry of Health, which recommends a minimum of six appointments during pregnancy, with at least one in the first trimester, two in the second trimester and three in the third, trimester with monthly intervals until the 28th week, biweekly until the 36th week and weekly until birth [22]. Using these recommendations, we defined adequate appointments as at least two until 20 weeks, three until 27 weeks, four until 30 weeks, five until 33 weeks, and six until delivery.

### Outcome

Our primary outcome was the number of respiratory-related hospitalisations in children under five, classified according to the International Classification of Diseases-10 (ICD-10, Chapter X, code range J00–J99). To evaluate the cause of hospitalisation, we used three-digit ICD-10 codes. If multiple hospitalisations occurred with overlapping periods and were recorded under different four-digit ICD-10 codes, they were consolidated into a single hospitalisation event, with the date of the first admission recorded as the event date.

The secondary outcomes were: 1) hospitalisation for specific blocks in the Chapter X: J00-J06 (Acute upper respiratory infections), J09-J18 (Influenza and Pneumonia), J20-J22 (Other acute lower respiratory infections); 2) deaths due to respiratory diseases (ICD-10 Chapter X); 3) all-cause mortality, the all-cause mortality outcome was included as previously recommended when evaluating recurrent events [23].

### Statistical analysis

We used entropy balancing, a type of inverse probability weighting, to estimate the average treatment effect size in the treated group, which captures the average differences in the outcomes in the preterm group. Entropy balancing ensures an exact balance of the covariate means while improving precision over traditional inverse probability weights [24]. We included maternal age at birth (linear and quadratic terms), marital status, parity, race/ethnicity, year of pregnancy, previous fetal loss, adequacy of number of prenatal appointments, years of schooling, state of residency, and municipality deprivation level (Brazilian deprivation index) in the model to estimate the entropy balancing weights.

To account for the terminal event of death when evaluating recurrent hospitalisations, Ghosh-Lin models were used to calculate Mean Ratios (MR) and 95% confidence intervals (CI). The Ghosh-Lin method is a semi‐parametric method that estimates the marginal mean of the cumulative number of recurrent events over time, acknowledging that death is a terminal event after which no further recurrent hospitalisations can be experienced [25]. We estimated the nonparametric mean cumulative function with variance calculated using the Lawless and Nadeau estimator.

We also estimated the Hazard Ratios (HR) and 95% CI using the cause-specific Cox model for death due to respiratory causes, as defined through ICD-10 codes; death from other causes was censored. Lastly, we estimated HR and 95% CI for all-cause mortality using the Cox model, as previously recommended when evaluating recurrent events [23].

We weighted all models using the inverse probability treatment weights for preterm to control for confounding. Missing data (< 5%) in the covariates was addressed in the weighting estimation by missing indicators [26].

We also conducted age-stratified analyses by dividing the datasets into subgroups based on age in days: 0–27 (neonatal period), 28–90, 91–365, 366–720 (1 year), 721–1095 (2 years), 1096–1460 (3 years), and 1461–1825 (4 years). These analyses included only live births at risk within each respective period. The analysis was conducted in R 4.3.1, utilising the WeightIt, survival, and mets packages.

## Results

**Fig. 1:**
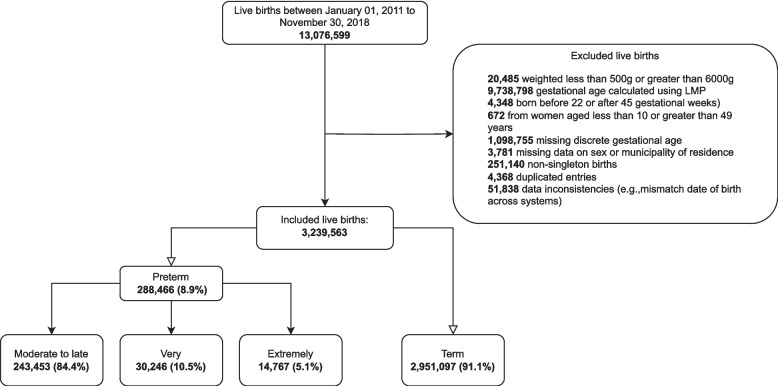
Selection of study participants. LMP = Last menstrual period

The study included 3,239,563 live births from 2011 to 2018; among these 288,466 (8.9%) were born preterm, and 243,453 (84.4%) were classified as moderate to late preterm (Fig. 1). The mothers of preterm and term live births were similar in terms of age, race/ethnicity, and geographic distribution, but those with a preterm birth had a lower proportion of an adequate number of prenatal appointments (174,536–61.7% versus 2,231,188–76.4%)(Table 1 and Supplementary Table 2). All variables achieved a satisfactory balance after weighting (Supplementary Figs. 1 to 4).

**Table 1:** Baseline characteristics of singleton live births

	**Term** ***N* = 2,951,097**	**Preterm** ***N* = 288,466**	**Overall** ***N* = 3,239,563**
Mother characteristic			
Age mother – years, median (IQR)	24 (20, 29)	24 (19, 30)	24 (20, 30)
Years of schooling			
None	15,944 (0.5)	1,776 (0.6)	17,720 (0.5)
1 to 3	85,533 (2.9)	9,378 (3.3)	94,911 (2.9)
4 to 7	678,241 (23.0)	70,407 (24.4)	748,648 (23.1)
8 to 11	1,963,752 (66.5)	185,960 (64.5)	2,149,712 (66.4)
≥ 12	190,005 (6.4)	18,847 (6.5)	208,852 (6.4)
Missing data	17,622 (0.6)	2,098 (0.7)	19,720 (0.6)
Race/ethnicity			
White	1,013,408 (34.3)	101,184 (35.1)	1,114,592 (34.4)
Black	244,639 (8.3)	24,266 (8.4)	268,905 (8.3)
Indigenous	15,278 (0.5)	1,346 (0.5)	16,624 (0.5)
Mixed	1,624,497 (55.0)	155,856 (54.0)	1,780,353 (55.0)
Asian	9,014 (0.3)	901 (0.3)	9,915 (0.3)
Missing data	44,261 (1.5)	4,913 (1.7)	49,174 (1.5)
Marital Status			
Single	32,610 (1.1)	3,530 (1.2)	36,140 (1.1)
Married	579,969 (19.7)	54,789 (19.0)	634,758 (19.6)
Divorced	1,654,045 (56.0)	164,197 (56.9)	1,818,242 (56.1)
Stable union	664,496 (22.5)	63,822 (22.1)	728,318 (22.5)
Widowed	4,795 (0.2)	488 (0.2)	5,283 (0.2)
Missing data	15,182 (0.5)	1,640 (0.6)	16,822 (0.5)
Deprivation Index-City			
1 (lowest deprivation)	623,404 (21.1)	61,203 (21.2)	684,607 (21.1)
2	663,452 (22.5)	64,127 (22.2)	727,579 (22.5)
3	787,349 (26.7)	73,686 (25.5)	861,035 (26.6)
4	556,922 (18.9)	53,770 (18.6)	610,692 (18.9)
5 (highest deprivation)	319,970 (10.8)	35,680 (12.4)	355,650 (11.0)
Adequate number of prenatal appointments	2,231,188 (76.4)	174,536 (61.7)	2,405,724 (75.1)
Missing data	27,125 (0.9)	5,393 (1.9)	32,518 (1.0)
Number of previous pregnancies			
0	1,866,754 (63.3)	174,366 (60.4)	2,041,120 (64.4)
≥ 1	1,022,395 (34.6)	108,326 (37.6)	1,130,721 (35.6)
Missing data	61,948 (2.1)	5,774 (2.0)	67,722 (2.1)
Previous fetal loss	509,346 (17.3)	61,009 (21.1)	570,355 (18.4)
Missing data	123,505 (4.2)	10,403 (3.6)	133,908 (4.1)
Gestational age (weeks), median (IQR)	39 (38—40)	35 (33—36)	39 (38—40)
Gestational age method			
Physical Exam	1,598,288 (54.2)	155,498 (53.9)	1,753,786 (54.1)
Ultrasonography	1,352,809 (45.8)	132,968 (46.1)	1,485,777 (45.9)
Live birth characteristics			
Preterm category			
moderate to late preterm (32 to 37 weeks)		243,451 (84.4)	243,451 (84.4)
very preterm (28 to less than 32 weeks)		30,246 (10.5)	30,246 (10.5)
extremely preterm (less than 28 weeks)		14,769 (5.1)	14,769 (5.1)
Sex- Male	1,509,774 (51.2)	152,266 (52.8)	1,662,040 (51.3)
Birth weight (g), median (IQR)	3,250 (2,970, 3,550)	2,350 (1,860, 2,722)	3,205 (2,890, 3,520)
Low birth weight (< 2500 g)	112,679 (3.8)	173,389 (60.1)	286,068 (8.8)
Weight for gestational age			
SGA	216,159 (7.3)	28,214 (9.8)	244,373 (7.5)
AGA	2,351,054 (79.7)	222,572 (77.2)	2,573,626 (79.4)
LGA	383,884 (13.0)	37,680 (13.1)	421,564 (13.0)
Congenital Anomaly	25,078 (0.8)	7,251 (2.5)	32,329 (1.0)
Missing data	17,797 (0.6)	2,520 (0.9)	20,317 (0.6)
Delayed antenatal care	697,108 (23.6)	70,600 (24.5)	767,708 (23.7)
Missing data	190,268 (6.4)	32,064 (11.1)	222,332 (6.9)
Apgar 5', median (IQR)	9.00 (9.00, 10.00)	9.00 (8.00, 10.00)	9.00 (9.00, 10.00)
Missing data	33,940 (1.2)	4,674 (1.6)	38,614 (1.2)
Low Apgar < 7	21,537 (0.7)	16,034 (5.6)	37,571 (1.2)
Missing data	33,940 (1.2)	4,674 (1.6)	38,614 (1.2)

*IQR* Interquartile range, Weight for gestational age was defined based on intergrowth charts and comprised: (1) small for gestational age (SGA) – i.e. birth weight < 10th percentile for sex and gestational age; (2) Appropriate for gestational age (AGA) – i.e. birthweight between 10 and 90th percentiles for sex and gestational age; (3) Large for gestational age (LGA) – i.e. birthweight > 90th percentile for sex and gestational age

### Respiratory-related hospitalisations

**Fig. 2:**
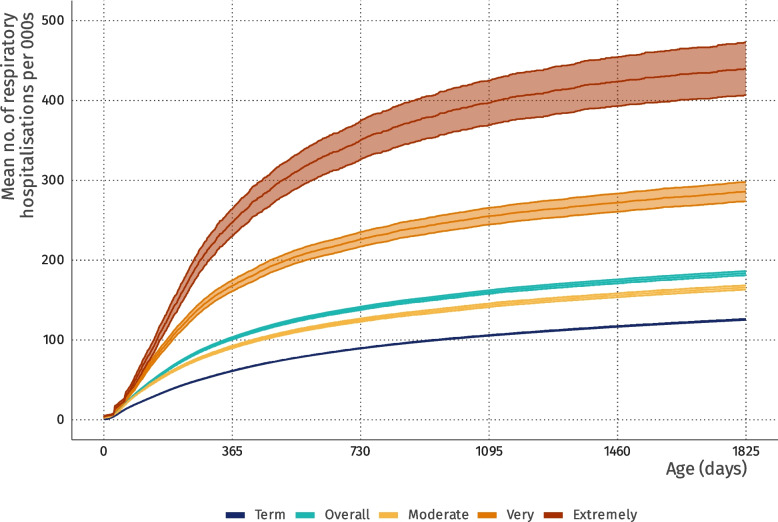
Mean cumulative count of respiratory hospitalisation curves and 95% confidence intervals stratified by term and preterm category*.* Overall represents all preterm categories combined

In the first four years of life, the preterm group experienced an average of 184 hospitalisations per 1000 children, compared with 126 hospitalisations per 1000 in the term group (Fig. 2). The estimated increase was 40% more respiratory-related hospitalisations (MR: 1.40, 95% CI: 1.38 to 1.42) comparing preterm to term children during the same period. The analysis by subgroup-specific ICD-10 blocks shows similar values for influenza and pneumonia (MR: 1.35; 1.32 to 1.37), for other acute lower respiratory infections (MR: 1.40; 1.37 to 1.44), and lower values for acute upper respiratory infections (MR: 1.26; 1.19 to 1.33) (Supplementary Table 4).

In the analysis by age, the highest increase in the number of hospitalisations comparing preterm and term children occurred during the period of 28–90 days, with an MR of 1.67 (95% CI: 1.63 to 1.72), and remained elevated between 91 and 365 days (MR: 1.65, 1.62 to 1.69). The risk gradually declined over time, reaching an MR of 1.18 (1.10 to 1.28) by the age of four years (Table 2 and Supplementary Table 3).

**Table 2:** Mean ratios for the number of respiratory-related hospitalisations and hazard ratios for respiratory-related mortality and all-cause mortality comparing preterm and term children

Category	Person years	No respiratory hospitalisations	Mean Ratio (95% CI)	No. respiratory deaths	Hazard Ratio—Respiratory Death (95% CI)
0–4 years					
Term	9,441,592	292,856	Reference	2024	Reference
Preterm					
Overall	854,531	40,072	1.40 (1.38 to 1.42)	783	3.95 (3.62 to 4.30)
Moderate to late	753,574	31,814	1.32 (1.30 to 1.34)	479	2.74 (2.48 to 3.03)
Very	80,174	5,892	1.97 (1.89 to 2.05)	202	10.76 (9.29 to 12.46)
Extremely	20,783	2,366	1.60 (1.49 to 1.72)	102	21.31 (17.42 to 26.06)
0–27 days					
Term	216,671	11,504	Reference	87	Reference
Preterm					
Overall	20,196	1,602	1.41 (1.34 to 1.49)	34	3.69 (2.46 to 5.55)
Moderate to late	17,617	1,353	1.41 (1.33 to 1.49)	21	2.61 (1.61 to 4.24)
Very	1,957	171	1.48 (1.27 to 1.73)	< 5	N/A
Extremely	622	78	1.37 (1.09 to 1.71)	9	N/A
28–90 days					
Term	497,049	44,869	Reference	559	Reference
Preterm					
Overall	45,518	7,056	1.68 (1.63 to 1.72)	261	4.66 (4.00 to 5.43)
Moderate to late	40,130	5,885	1.58 (1.54 to 1.63)	157	3.18 (2.65 to 3.81)
Very	4,252	882	2.26 (2.11 to 2.42)	69	13.00 (10.08 to 16.77)
Extremely	1,136	289	2.62 (2.32 to 2.95)	35	25.83 (18.28 to 36.50)
91–365 days					
Term	2,025,405	113,189	Reference	771	Reference
Preterm					
Overall	183,921	17,174	1.65 (1.62 to 1.69)	345	4.58 (4.02 to 5.23)
Moderate to late	162,521	13,135	1.43 (1.40 to 1.46)	195	2.94 (2.50 to 3.45)
Very	17,012	2,884	3.02 (2.88 to 3.17)	105	14.84 (12.06 to 18.26)
Extremely	4,389	1,155	4.58 (4.24 to 4.96)	45	25.44 (18.77 to 34.47)
1 year					
Term	2,326,565	66,898	Reference	383	Reference
Preterm					
Overall	209,739	7,992	1.34 (1.30 to 1.37)	92	2.46 (1.95 to 3.10)
Moderate to late	185,335	6,322	1.20 (1.16 to 1.23)	66	2.01 (1.54 to 2.61)
Very	19,414	1,146	2.08 (1.93 to 2.24)	16	4.51 (2.73 to 7.47)
Extremely	4,990	524	3.63 (3.22 to 4.09)	10	11.06 (5.88 to 20.80)
2 years					
Term	1,919,681	31,069	Reference	129	Reference
Preterm					
Overall	172,584	3,518	1.26 (1.21 to 1.32)	36	3.12 (2.14 to 4.55)
Moderate to late	152,191	2,848	1.16 (1.11 to 1.21)	28	2.75 (1.81 to 4.16)
Very	16,222	474	1.81 (1.62 to 2.03)	5	N/A
Extremely	4,171	196	2.87 (2.39 to 3.45)	< 5	N/A
3 years					
Term	1,469,740	16,621	Reference	75	Reference
Preterm					
Overall	132,408	1,795	1.21 (1.14 to 1.28)	9	N/A
Moderate to late	116,568	1,493	1.14 (1.08 to 1.22)	7	N/A
Very	12,591	214	1.52 (1.28 to 1.81)	< 5	N/A
Extremely	3,249	88	2.38 (1.83 to 3.10)	< 5	N/A
4 years					
Term	986,481	8,706	Reference	20	Reference
Preterm					
Overall	90,165	935	1.18 (1.10 to 1.28)	6	N/A
Moderate to late	79,213	778	1.12 (1.03 to 1.22)	5	N/A
Very	8,727	121	1.59 (1.29 to 1.95)	< 5	N/A
Extremely	2,225	36	1.82 (1.19 to 2.80)	< 5	N/A

N/A: To ensure reliable estimates, only periods with at least 10 events in each group were estimated

When stratified by gestational age, the burden of respiratory-related hospitalisations increased with the degree of prematurity. Compared with term children, moderate to late preterm children had 32% more hospitalisations (MR: 1.32, 95% CI: 1.30 to 1.34), whereas very preterm and extremely preterm children had 97 (MR: 1.97, 1.89 to 2.05) and 60 (MR: 1.60, 1.49 to 1.72) more hospitalisations, respectively. The highest hospitalisation rates for very preterm infants and extremely preterm infants occurred between 91 and 365 days of life, with MRs of 3.02 (2.88 to 3.17) and 4.58 (4.24 to 4.96), respectively (Fig. 2 and Table 2). At the age of 5 years, on average, 430 respiratory hospitalisations occurred per 1,000 extremely preterm children. (Fig. 2).

### Respiratory-related mortality

Preterm children faced a higher rate of respiratory-related mortality compared to term children. The overall HR for respiratory-related mortality in preterm children under five was 3.95 (95% CI: 3.62–4.30). The risk was highest between 28 and 90 days of life, with an HR of 4.66 (95% CI: 4.00–5.43). (Table 2 and Supplementary Table 3).

The rates of respiratory-related mortality increased with the degree of prematurity, showing a clear dose–response relationship over the first four years of life. Compared with term children, moderate to late preterm children had an HR of 2.74 (95% CI: 2.48–3.03) for respiratory-related mortality. For very preterm infants, the HR was 10.76 (95% CI: 9.29–12.46), and for extremely preterm infants, the HR reached 21.31 (95% CI: 17.42–26.06). (Table 2).

The timing of the highest mortality risk varied by gestational age. For moderate to late and extremely preterm children, the highest rates occurred between 28 and 90 days (HRs: 2.94; 95% CI: 2.50 to 3.45, and 25.83; 95% CI: 18.28 to 36.50, respectively). However, for very preterm children, it was between 91 and 365 days (HR: 14.84; 12.06 to 18.26). (Table 2).

## Discussion

In this nationwide cohort study, we found that children born preterm face an increased risk of respiratory-related hospitalisation and mortality compared with their term-born counterparts. The risk was particularly pronounced during the first year of life and exhibited a clear dose–response relationship, with the highest risks observed among children born at earlier gestational ages. Specifically, children born before 28 weeks of gestation had the highest mortality rates, with neonatal respiratory-related mortality exceeding 21 times that of term-born children. Similarly, the number of respiratory-related hospitalisations increased with decreasing gestational age, with extremely preterm infants experiencing nearly three times more hospitalisations between 91 and 365 days of life compared to term infants.

These findings align with a growing body of evidence highlighting the long-term respiratory morbidity associated with preterm birth [27, 28]. Preterm infants often experience disrupted alveolar development, small airway disease, and gas trapping, which can lead to structural lung abnormalities and impaired lung function throughout their lives [27, 29]. While much of the existing research has focused on bronchopulmonary dysplasia (BPD) in extremely preterm infants, it is increasingly clear that even late preterm infants and those without BPD remain at risk for significant respiratory disease later in life [27, 29, 30]. This includes conditions such as transient tachypnea of the newborn, respiratory distress syndrome, pneumonia, and pulmonary hypertension, all of which occur at higher rates in preterm infants compared to term infants [16, 27, 31]. Additionally, preterm infants, particularly those born very premature or with BPD, are more vulnerable to severe lower respiratory tract infections, which often require frequent hospitalisations and intensive care [10, 32–35]. Moreover, our study observed increased vulnerability across distinct respiratory subgroups, including acute upper respiratory infections, influenza and pneumonia, and other acute lower respiratory infections, indicating increased vulnerability in preterm children to different conditions.

A key strength of this study is its large sample size, which enhances the precision and generalisability of our findings. By employing a robust methodological approach that accounts for recurrent events and competing risks, we were able to provide a more accurate estimation of the burden of respiratory complications in preterm children. We also estimated the effect of preterm birth on overall mortality, providing a more comprehensive and transparent assessment of the morbidity associated with preterm birth, as the interpretation of hospitalisation rates must consider the high all-cause mortality among preterm infants, particularly the extremely preterm group. Extremely preterm infants faced the highest mortality rate, with a neonatal mortality rate 146 times higher than that of term infants. (Supplementary Table 3) This high mortality risk likely led to a reduction in the observed hospitalisation rates for extremely preterm infants, as many infants died before they could be hospitalised. For example, the MR for respiratory-related hospitalisations in extremely preterm infants during the neonatal period was 1.37, which is lower than the MR for very preterm infants (MR: 1.48).

Our findings are consistent with those of previous studies that reported increased risks of respiratory-related hospitalisations and mortality in preterm children, although they evaluated only the first hospitalisation due to respiratory diseases. For example, studies have shown that late preterm infants have a 1.3 to 2.0 times higher risk of respiratory-related hospitalisations compared to term infants, depending on gestational age [16, 17, 28, 36]. Similarly, our mortality rates for both all-cause and respiratory-related deaths are comparable to those reported in international studies, further validating our results [2, 37]. Furthermore, a meta-analysis focused on the impact of RSV on preterm infants reported a hospitalisation rate of 34 per 1000 preterm infants under one year for high-income countries, whereas for upper-middle-income countries it was 68 [8]. The meta-analysis found an overall admission rate per 1000 of 37 for preterm and 16 for term infants [8]. Our study, which assessed all respiratory-related causes, the rate reached 102 hospitalisations per 1000 preterm children in the first year of life and 61 for the term children. While our broader inclusion criteria likely explain the higher absolute numbers, the persistent, significant gap between preterm and term cohorts, regardless of setting, reinforces their vulnerability.

However, our study is not without limitations. Firstly, there is potential for residual confounding due to the unavailability of data on specific variables, such as the quality and access to healthcare. Secondly, our study is susceptible to linkage errors; nonetheless, we expected that these errors would occur non-differentially, which could lead to an underestimation of the association. Thirdly, the CIDACS Birth Cohort encompasses only the most socially vulnerable live births in Brazil, which may restrict the generalisability of our findings to less vulnerable populations. Fourthly, we excluded many live births due to missing data regarding gestational age at birth in weeks. This missing data is likely attributable to the transition in 2011 when the SINASC system began recording gestational age by week instead of intervals, rather than suggesting systematic bias or confounding. Fifthly, despite the substantial sample size, specific risk periods in our stratified analyses had a low number of events, precluding precise estimates. Finally, we restricted our analysis to respiratory diseases classified under Chapter X of the ICD-10, which may have excluded pertinent respiratory conditions categorised elsewhere, such as those originating in the perinatal period (Chapter XVI).

Despite these limitations, our study provides critical insights into the burden of respiratory morbidity and mortality in preterm children in an LMIC setting. These findings underscore the importance of recognising preterm birth as a key risk factor for both morbidity and mortality, which can inform the development of targeted health strategies. Interventions to address complications from preterm birth and preventive measures like immunisation against respiratory infections, including respiratory syncytial virus, are essential for improving respiratory health outcomes in preterm infants.

In conclusion, preterm newborns face a significantly higher risk of respiratory illnesses compared to full-term children, particularly during their first year of life. This insight highlights critical periods of vulnerability, which can inform the development of targeted health strategies to address the challenges associated with premature birth. Recognising preterm birth as a key risk factor for both respiratory mortality and morbidity is essential for guiding preventive approaches and improving health outcomes for preterm infants, especially in resource-limited settings.

## Supplementary Information

Supplementary Material 1.

## Data Availability

The relevant raw data are available upon reasonable request to the Centro de Integração de Dados e Conhecimentos para a Saúde (CIDACS). Any person who wishes to receive authorisation must: (1) be affiliated to CIDACS or be accepted as collaborators; (2) present a detailed research project together with approval by an appropriate Brazilian institutional research ethics committee; (3) provide a clear data plan restricted to the objectives of the proposed study and a summary of the analyses plan intended to guide the linkage and data extraction of the relevant set of records and variables; (4) sign terms of responsibility regarding the access and use of data; and (5) perform the analyses of datasets provided using the CIDACS data environment, a safe and secure infrastructure that provides remote access to de-identified or anonymised datasets and analysis tools. For more information: https://cidacs.bahia.fiocruz.br/.
