# Supplementary material for: Nationwide study of respiratory-related hospitalisations and deaths in preterm children in Brazil: a registry-based study
Source: Respir Res. 2025 Dec 13;27:17. doi: 10.1186/s12931-025-03449-6 (PMC12805753; doi:10.1186/s12931-025-03449-6)
Supplement: Supplementary file 1 — Supplementary Material 1. [file 12931_2025_3449_MOESM1_ESM.docx]

# Supplementary Material

Table of Contents

[Supplementary Material 1](#_Toc213088537)

[Supplementary Figure 1: Love plots representing the effect of the weighting in terms of standardised mean differences (SMD) among the weighted variables in preterm vs term children. 2](#_Toc213088538)

[Supplementary Figure 2: Love plots representing the effect of the weighting in terms of standardised mean differences (SMD) among the weighted variables in moderate to late preterm vs term children. 3](#_Toc213088539)

[Supplementary Figure 3: Love plots representing the effect of the weighting in terms of standardised mean differences (SMD) among the weighted variables in very preterm vs term children. 4](#_Toc213088540)

[Supplementary Figure 4: Love plots representing the effect of the weighting in terms of standardised mean differences (SMD) among the weighted variables in extreme preterm vs term children. 5](#_Toc213088541)

[Supplementary Table 1: Distribution of weights for gestational, median weight and low birth weight proportion of term and preterm live births across the different estimator methods for gestational age. 6](#_Toc213088542)

[Supplementary Table 2: Baseline characteristics of singleton live births 7](#_Toc213088543)

[Supplementary Table 3: Mean ratios for the number of respiratory-related hospitalisations and hazard ratios for respiratory-related mortality and all-cause mortality comparing preterm and term children. 10](#_Toc213088544)

[Supplementary Table 4: Mean ratios for the number of respiratory-related hospitalisations and hazard ratios for respiratory-related mortality comparing preterm and term children for specific ICD-10 blocks. 12](#_Toc213088545)


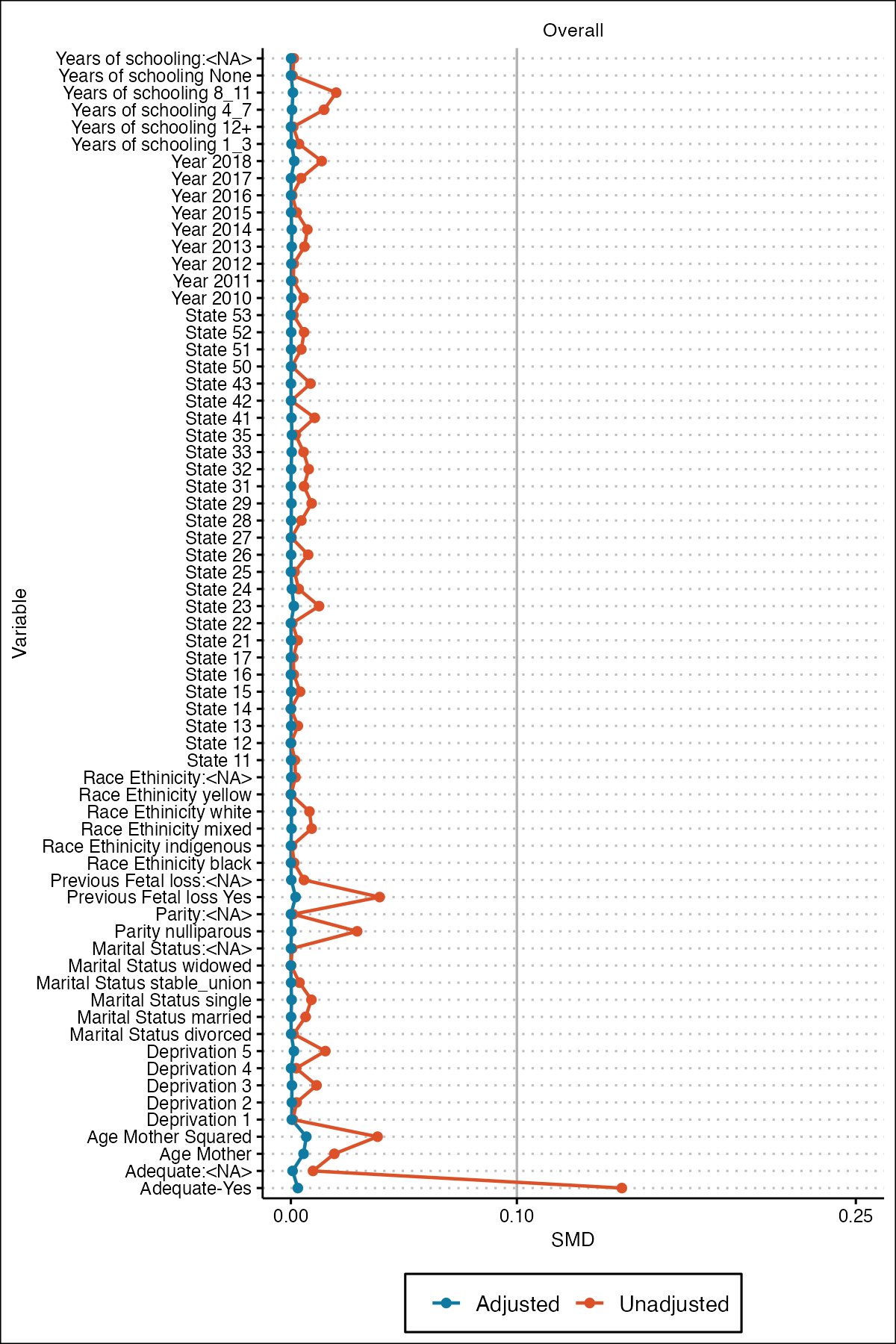


# Supplementary Figure 1: Love plots representing the effect of the weighting in terms of standardised mean differences (SMD) among the weighted variables in preterm vs term children.


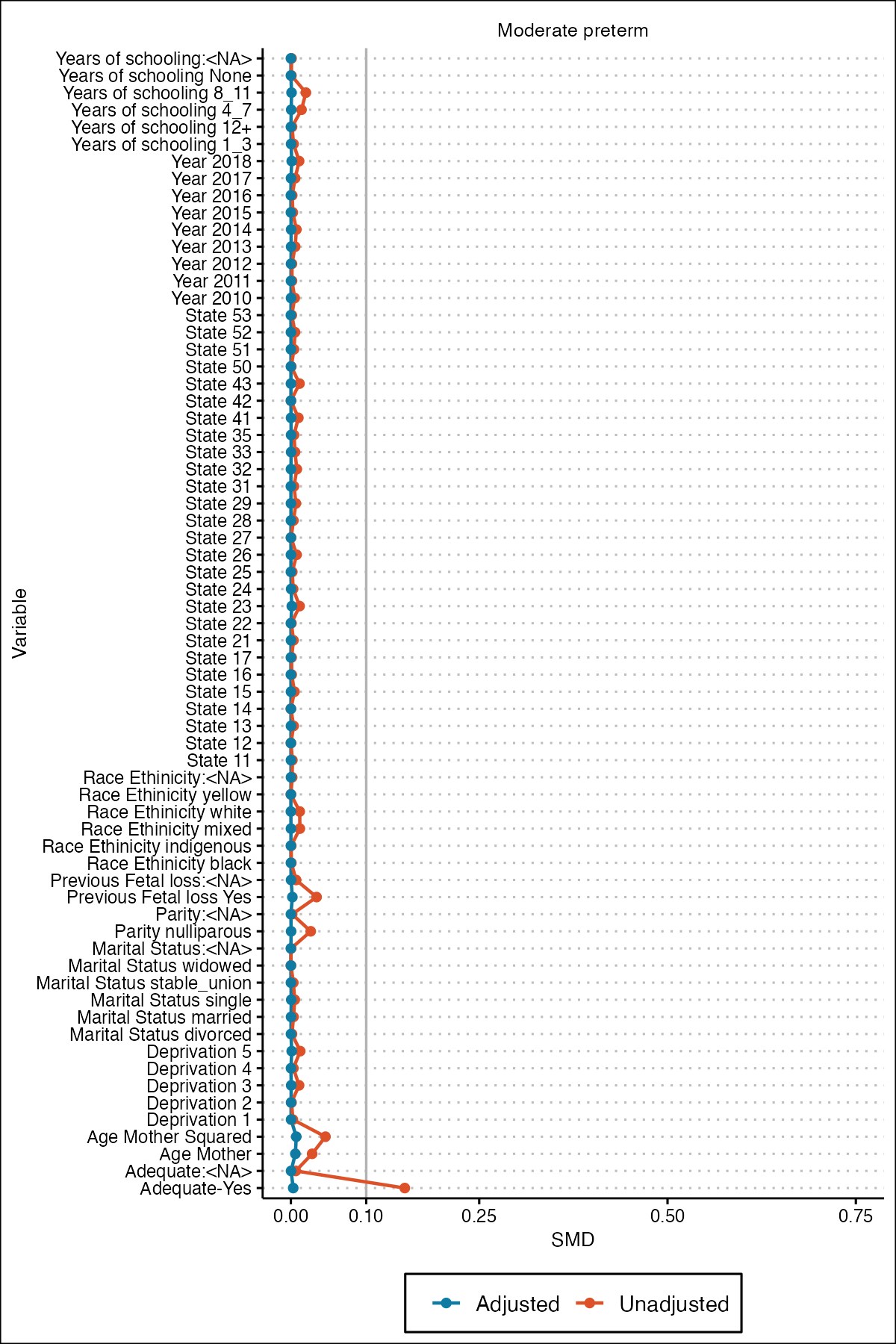


# Supplementary Figure 2: Love plots representing the effect of the weighting in terms of standardised mean differences (SMD) among the weighted variables in moderate to late preterm vs term children.


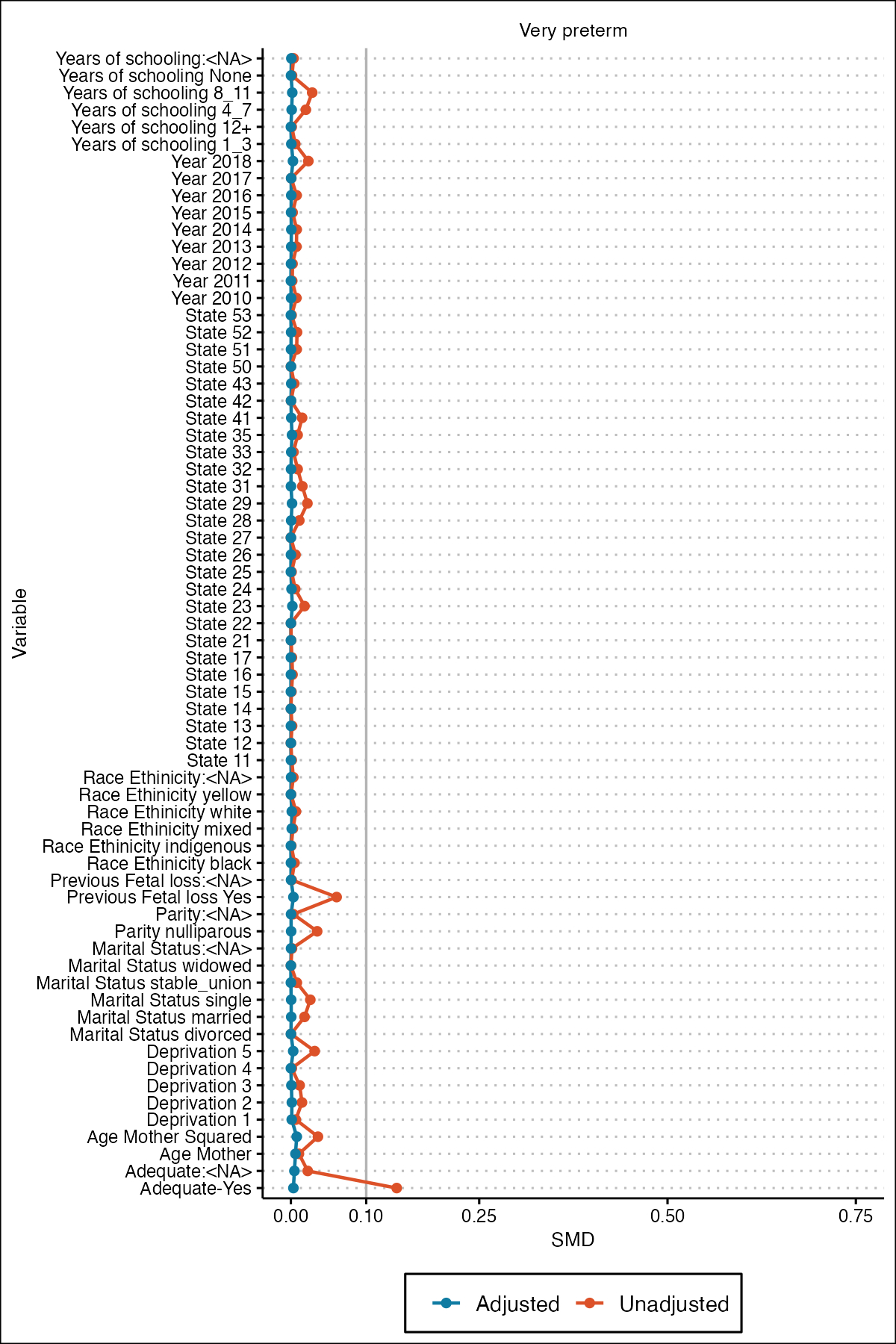


# Supplementary Figure 3: Love plots representing the effect of the weighting in terms of standardised mean differences (SMD) among the weighted variables in very preterm vs term children.


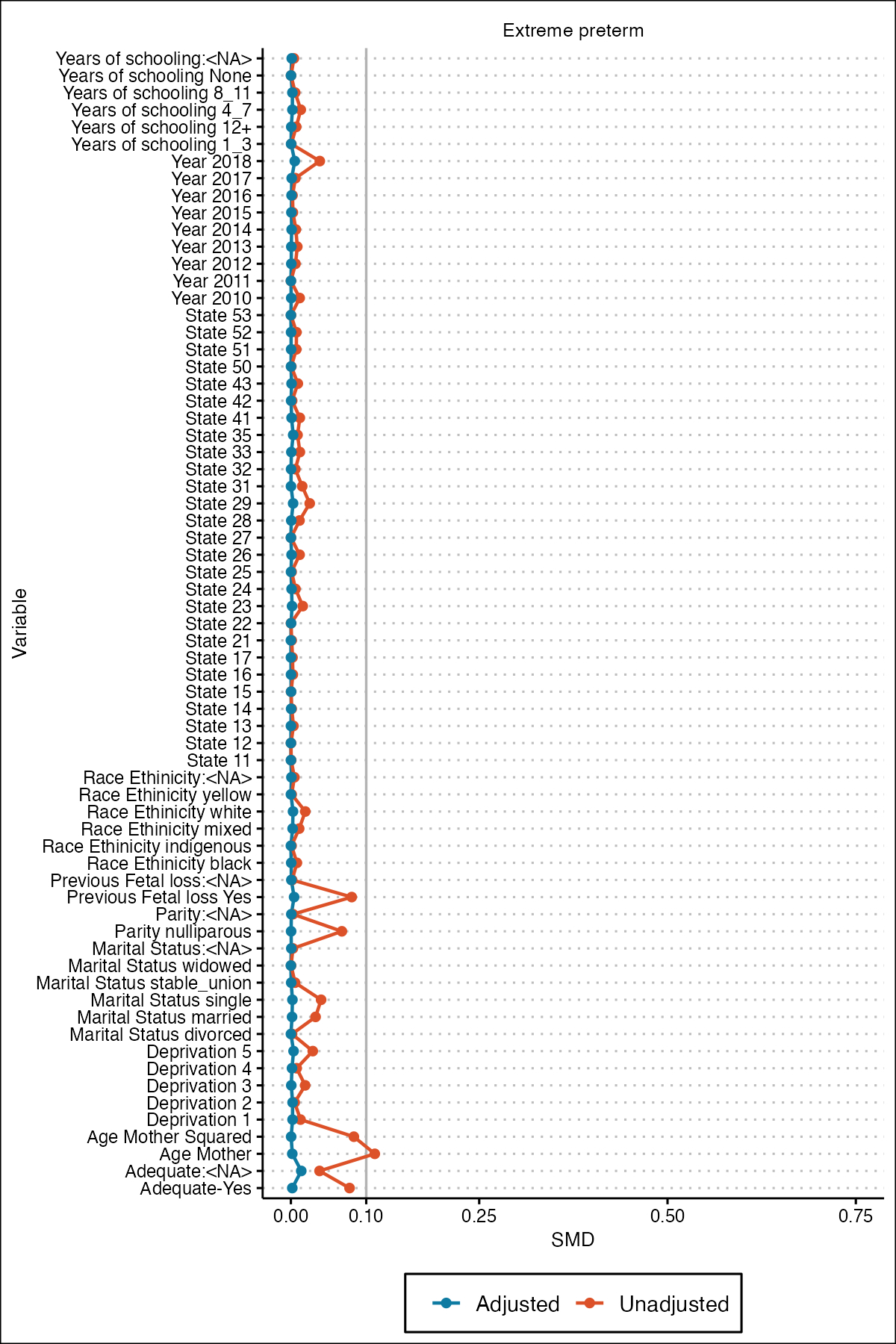


# Supplementary Figure 4: Love plots representing the effect of the weighting in terms of standardised mean differences (SMD) among the weighted variables in extreme preterm vs term children.

# Supplementary Table 1: Distribution of weights for gestational, median weight and low birth weight proportion of term and preterm live births across the different estimator methods for gestational age.

|  | **Term** | **Preterm** | **Total** |
| --- | --- | --- | --- |
| **Weight for gestational age** |  |  |  |
| LMP + Other methods |  |  |  |
| SGA | 896,725 (8.6) | 75,074 (5.9) | 971,799 (8.3) |
| AGA | 8,028,895 (77.4) | 693,887 (54.9) | 8,722,782 (74.9) |
| LGA | 1,452,246 (14.0) | 494,385 (39.1) | 1,946,631 (16.7) |
|  |  |  |  |
| Other methods |  |  |  |
| SGA | 216,159 (7.3) | 28,214 (9.8) | 244,373 (7.5) |
| AGA | 2,351,054 (79.7) | 222,572 (77.2) | 2,573,626 (79.4) |
| LGA | 383,884 (13.0) | 37,680 (13.1) | 421,564 (13.0) |
|  |  |  |  |
| Only LMP |  |  |  |
| SGA | 680,566 (9.2) | 46,860 (4.8) | 727,426 (8.7) |
| AGA | 5,677,841 (76.5) | 471,315 (48.3) | 6,149,156 (73.2) |
| LGA | 1,068,362 (14.4) | 456,705 (46.8) | 1,525,067 (18.2) |
|  |  |  |  |
|  |  |  |  |
| **Birth weight (g)** |  |  |  |
| LMP + Other methods | 3,270 (2,990 - 3,565) | 2,740 (2,235 - 3,180) | 3,230 (2,925 - 3,540) |
| Other methods | 3,250 (2,970 - 3,550) | 2,350 (1,860 -2,722) | 3,205 (2,890 - 3,520) |
| Only LMP | 3,275 (3,000 - 3,570) | 2,865 (2,385 - 3,265) | 3,240 (2,940 - 3,550) |
|  |  |  |  |
| **Low birth weight (<2500g)** |  |  |  |
| LMP + Other methods | 377,282 (3.6) | 461,454 (36.5) | 838,736 (7.2) |
| Other methods | 112,679 (3.8) | 173,389 (60.1) | 286,068 (8.8) |
| Only LMP | 264603 (3.6) | 288,065 (29.6) | 552,668 (6.6) |

The distribution using only other methods, such as physical exam and ultrasonography, is closer to a normal distribution, with similar rates of large for gestational age among term and preterm live births, and a higher proportion of small for gestational age in the preterm group. Meanwhile, the distribution of the total sample and those with gestational age estimated only through last menstrual period is skewed toward a higher proportion of large for gestational age at 39.1% and 46.8%, respectively. The median weight of preterm live births in the samples, including those with gestational age estimated using LMP, is also above 2,500g. This pattern likely indicates misclassification of gestational age estimated using the last menstrual period.[1]

# Supplementary Table 2: Baseline characteristics of singleton live births

| **Characteristic** | **Term N = 2,951,097** | **Preterm N = 288,466** | **Overall N = 3,239,563** |
| --- | --- | --- | --- |
| **Age mother - group** |  |  |  |
| 10-17 | 323,620 (11.0) | 40,021 (13.9) | 363,641 (11.2) |
| 18-24 | 1,209,107 (41.0) | 109,157 (37.8) | 1,318,264 (40.7) |
| 25-29 | 680,729 (23.1) | 59,144 (20.5) | 739,873 (22.8) |
| 30-34 | 448,221 (15.2) | 44,348 (15.4) | 492,569 (15.2) |
| 35-49 | 289,420 (9.8) | 35,796 (12.4) | 325,216 (10.0) |
| **Age mother – years, median (IQR)** | 24 (20, 29) | 24 (19, 30) | 24 (20, 30) |
| **Years of schooling** |  |  |  |
| None | 15,944 (0.5) | 1,776 (0.6) | 17,720 (0.5) |
| 1 to 3 | 85,533 (2.9) | 9,378 (3.3) | 94,911 (2.9) |
| 4 to 7 | 678,241 (23.0) | 70,407 (24.4) | 748,648 (23.1) |
| 8 to 11 | 1,963,752 (66.5) | 185,960 (64.5) | 2,149,712 (66.4) |
| ≥12 | 190,005 (6.4) | 18,847 (6.5) | 208,852 (6.4) |
| Missing data | 17,622 (0.6) | 2,098 (0.7) | 19,720 (0.6) |
| **Race/ethnicity** |  |  |  |
| White | 1,013,408 (34.3) | 101,184 (35.1) | 1,114,592 (34.4) |
| Black | 244,639 (8.3) | 24,266 (8.4) | 268,905 (8.3) |
| Indigenous | 15,278 (0.5) | 1,346 (0.5) | 16,624 (0.5) |
| Mixed | 1,624,497 (55.0) | 155,856 (54.0) | 1,780,353 (55.0) |
| Asian | 9,014 (0.3) | 901 (0.3) | 9,915 (0.3) |
| Missing data | 44,261 (1.5) | 4,913 (1.7) | 49,174 (1.5) |
| **Marital Status** |  |  |  |
| Single | 32,610 (1.1) | 3,530 (1.2) | 36,140 (1.1) |
| Married | 579,969 (19.7) | 54,789 (19.0) | 634,758 (19.6) |
| Divorced | 1,654,045 (56.0) | 164,197 (56.9) | 1,818,242 (56.1) |
| Stable union | 664,496 (22.5) | 63,822 (22.1) | 728,318 (22.5) |
| Widowed | 4,795 (0.2) | 488 (0.2) | 5,283 (0.2) |
| Missing data | 15,182 (0.5) | 1,640 (0.6) | 16,822 (0.5) |
| **Geographic Region** |  |  |  |
| North | 201,785 (6.8) | 18,231 (6.3) | 220,016 (6.8) |
| Northeast | 545,173 (18.5) | 60,329 (20.9) | 605,502 (18.7) |
| Southeast | 1,429,189 (48.4) | 138,097 (47.9) | 1,567,286 (48.4) |
| South | 579,067 (19.6) | 56,127 (19.5) | 635,194 (19.6) |
| Central-west | 195,883 (6.6) | 15,682 (5.4) | 211,565 (6.5) |
| **Deprivation Index-City** |  |  |  |
| 1 (lowest deprivation) | 623,404 (21.1) | 61,203 (21.2) | 684,607 (21.1) |
| 2 | 663,452 (22.5) | 64,127 (22.2) | 727,579 (22.5) |
| 3 | 787,349 (26.7) | 73,686 (25.5) | 861,035 (26.6) |
| 4 | 556,922 (18.9) | 53,770 (18.6) | 610,692 (18.9) |
| 5 (highest deprivation) | 319,970 (10.8) | 35,680 (12.4) | 355,650 (11.0) |
| **Number of prenatal appointments** | |  |  |
| None | 65,145 (2.2) | 15,044 (5.2) | 80,189 (2.5) |
| 1 to 3 | 228,960 (7.8) | 50,061 (17.4) | 279,021 (8.6) |
| 4 to 6 | 771,292 (26.1) | 110,783 (38.4) | 882,075 (27.2) |
| ≥7 | 1,858,575 (63.0) | 107,185 (37.2) | 1,965,760 (60.7) |
| Missing data | 27,125 (0.9) | 5,393 (1.9) | 32,518 (1.0) |
| **Adequate number of prenatal appointments** | 2,231,188 (76.4) | 174,536 (61.7) | 2,405,724 (75.1) |
| **Number of previous pregnancies** | |  |  |
| 0 | 1,866,754 (63.3) | 174,366 (60.4) | 2,041,120 (64.4) |
| ≥1 | 1,022,395 (34.6) | 108,326 (37.6) | 1,130,721 (35.6) |
| Missing data | 61,948 (2.1) | 5,774 (2.0) | 67,722 (2.1) |
| **Previous fetal loss** | 509,346 (17.3) | 61,009 (21.1) | 570,355 (18.4) |
| Missing data | 123,505 (4.2) | 10,403 (3.6) | 133,908 (4.1) |
| **Gestational age, median (IQR)** | 39.00 (38.00 - 40.00) | 35.00 (33.00 - 36.00) | 39.00 (38.00 - 40.00) |
| **Gestational age method** |  |  |  |
| Physical Exam | 1,598,288 (54.2) | 155,498 (53.9) | 1,753,786 (54.1) |
| Ultrasonography | 1,352,809 (45.8) | 132,968 (46.1) | 1,485,777 (45.9) |
| **Preterm category** |  |  |  |
| moderate to late preterm (32 to 37 weeks) | | 243,451 (84.4) | 243,451 (84.4) |
| very preterm (28 to less than 32 weeks) | | 30,246 (10.5) | 30,246 (10.5) |
| extremely preterm (less than 28 weeks) | | 14,769 (5.1) | 14,769 (5.1) |
| **Sex of live birth** |  |  |  |
| Male | 1,509,774 (51.2) | 152,266 (52.8) | 1,662,040 (51.3) |
| Female | 1,441,323 (48.8) | 136,200 (47.2) | 1,577,523 (48.7) |
| **Year of birth** |  |  |  |
| 2011 | 127,493 (4.3) | 12,766 (4.4) | 140,259 (4.3) |
| 2012 | 284,001 (9.6) | 28,911 (10.0) | 312,912 (9.7) |
| 2013 | 383,701 (13.0) | 37,331 (12.9) | 421,032 (13.0) |
| 2014 | 474,401 (16.1) | 45,161 (15.7) | 519,562 (16.0) |
| 2015 | 490,522 (16.6) | 46,295 (16.0) | 536,817 (16.6) |
| 2016 | 402,268 (13.6) | 39,175 (13.6) | 441,443 (13.6) |
| 2017 | 416,982 (14.1) | 41,562 (14.4) | 458,544 (14.2) |
| 2018 | 371,729 (12.6) | 37,265 (12.9) | 408,994 (12.6) |
| **Birth weight (g), median (IQR)** | 3,250 (2,970, 3,550) | 2,350 (1,860, 2,722) | 3,205 (2,890, 3,520) |
| **Low birth weight (<2500g)** | 112,679 (3.8) | 173,389 (60.1) | 286,068 (8.8) |
| **Weight for gestational age** |  |  |  |
| SGA | 216,159 (7.3) | 28,214 (9.8) | 244,373 (7.5) |
| AGA | 2,351,054 (79.7) | 222,572 (77.2) | 2,573,626 (79.4) |
| LGA | 383,884 (13.0) | 37,680 (13.1) | 421,564 (13.0) |
| **Congenital Anomaly** | 25,078 (0.8) | 7,251 (2.5) | 32,329 (1.0) |
| Missing data | 17,797 (0.6) | 2,520 (0.9) | 20,317 (0.6) |
| **Delayed antenatal care** | 697,108 (23.6) | 70,600 (24.5) | 767,708 (23.7) |
| Missing data | 190,268 (6.4) | 32,064 (11.1) | 222,332 (6.9) |
| **Apgar 5', median (IQR)** | 9.00 (9.00, 10.00) | 9.00 (8.00, 10.00) | 9.00 (9.00, 10.00) |
| Missing data | 33,940 (1.2) | 4,674 (1.6) | 38,614 (1.2) |
| **Low Apgar <7** | 21,537 (0.7) | 16,034 (5.6) | 37,571 (1.2) |
| Missing data | 33,940 (1.2) | 4,674 (1.6) | 38,614 (1.2) |

# Supplementary Table 3: Mean ratios for the number of respiratory-related hospitalisations and hazard ratios for respiratory-related mortality and all-cause mortality comparing preterm and term children.

| **Category** | | **No. children** | **Person years** | **No respiratory hospitalisations** | **Mean Ratio (95% CI)** | **No. respiratory deaths** | **Hazard Ratio - Respiratory Death (95% CI)** | **No. deaths** | **Hazard Ratio - All cause mortality (95% CI)** |  |  |  |  |  |  |  |  |
| --- | --- | --- | --- | --- | --- | --- | --- | --- | --- | --- | --- | --- | --- | --- | --- | --- | --- |
| **Term** | | 2951097 | 9441592 | 292856 | Ref | 2024 | Ref | 18357 | Ref |  |  |  |  |  |  |  |  |
| **Preterm** | | 288466 | 854531 | 40072 | 1.40 (1.38 to 1.42) | 783 | 3.95 (3.62 to 4.30) | 22485 | 11.99 (11.74 to 12.24) |  |  |  |  |  |  |  |  |
| *Moderate to late* | | 243453 | 753574 | 31814 | 1.32 (1.30 to 1.34) | 479 | 2.74 (2.48 to 3.03) | 8001 | 4.92 (4.79 to 5.06) |  |  |  |  |  |  |  |  |
| *Very* | | 30246 | 80174 | 5892 | 1.97 (1.89 to 2.05) | 202 | 10.76 (9.29 to 12.46) | 5904 | 31.93 (30.95 to 32.94) |  |  |  |  |  |  |  |  |
| *Extremely* | | 14767 | 20783 | 2366 | 1.60 (1.49 to 1.72) | 102 | 21.31 (17.42 to 26.06) | 8580 | 146.62 (142.41 to 150.95) |  |  |  |  |  |  |  |  |
| **0-27** | | | | | | | | | |  |  |  |  |  |  |  |  |
| **Term** | 2951097 | | 216671 | 11504 | Ref | 87 | Ref | 7079 | Ref |  |  |  |  |  |  |  |  |
| **Preterm** | 288466 | | 20196 | 1602 | 1.41 (1.34 to 1.49) | 34 | 3.69 (2.46 to 5.55) | 17000 | 22.95 (22.28 to 23.65) |  |  |  |  |  |  |  |  |
| *Moderate to late* | 243453 | | 17617 | 1353 | 1.41 (1.33 to 1.49) | 21 | 2.61 (1.61 to 4.24) | 4917 | 7.75 (7.47 to 8.06) |  |  |  |  |  |  |  |  |
| *Very* | 30246 | | 1957 | 171 | 1.48 (1.27 to 1.73) | <5 | N/A | 4540 | 59.21 (56.90 to 61.61) |  |  |  |  |  |  |  |  |
| *Extremely* | 14767 | | 622 | 78 | 1.37 (1.09 to 1.71) | 9 | N/A | 7543 | 267.53 (258.05 to 277.36) |  |  |  |  |  |  |  |  |
| **28-90** | | | | | | | | | |  |  |  |  |  |  |  |  |
| **Term** | | 2915024 | 497049 | 44869 | Ref | 559 | Ref | 3308 | Ref |  |  |  |  |  |  |  |  |
| **Preterm** | | 268609 | 45518 | 7056 | 1.68 (1.63 to 1.72) | 261 | 4.66 (4.00 to 5.43) | 2794 | 8.23 (7.81 to 8.68) |  |  |  |  |  |  |  |  |
| *Moderate to late* | | 236067 | 40130 | 5885 | 1.58 (1.54 to 1.63) | 157 | 3.18 (2.65 to 3.81) | 1328 | 4.45 (4.17 to 4.75) |  |  |  |  |  |  |  |  |
| *Very* | | 25413 | 4252 | 882 | 2.26 (2.11 to 2.42) | 69 | 13.00 (10.08 to 16.77) | 772 | 23.82 (21.97 to 25.83) |  |  |  |  |  |  |  |  |
| *Extremely* | | 7129 | 1136 | 289 | 2.62 (2.32 to 2.95) | 35 | 25.83 (18.28 to 36.50) | 694 | 82.12 (75.42 to 89.41) |  |  |  |  |  |  |  |  |
| **91-365** | | | | | | | | | |  |  |  |  |  |  |  |  |
| **Term** | | 2847010 | 2025405 | 113189 | Ref | 771 | Ref | 4206 | Ref |  |  |  |  |  |  |  |  |
| **Preterm** | | 259830 | 183921 | 17174 | 1.65 (1.62 to 1.69) | 345 | 4.58 (4.02 to 5.23) | 1905 | 4.52 (4.28 to 4.78) |  |  |  |  |  |  |  |  |
| *Moderate to late* | | 229434 | 162521 | 13135 | 1.43 (1.40 to 1.46) | 195 | 2.94 (2.50 to 3.45) | 1176 | 3.17 (2.96 to 3.38) |  |  |  |  |  |  |  |  |
| *Very* | | 24106 | 17012 | 2884 | 3.02 (2.88 to 3.17) | 105 | 14.84 (12.06 to 18.26) | 459 | 11.56 (10.48 to 12.75) |  |  |  |  |  |  |  |  |
| *Extremely* | | 6290 | 4389 | 1155 | 4.58 (4.24 to 4.96) | 45 | 25.44 (18.77 to 34.47) | 270 | 27.20 (24.01 to 30.82) |  |  |  |  |  |  |  |  |
| **1 year** | | | | | | | | | |  |  |  |  |  |  |  |  |
| **Term** | | 2532640 | 2326565 | 66898 | Ref | 383 | Ref | 1924 | Ref |  |  |  |  |  |  |  |  |
| **Preterm** | | 228725 | 209739 | 7992 | 1.34 (1.30 to 1.37) | 92 | 2.46 (1.95 to 3.10) | 473 | 2.53 (2.28 to 2.80) |  |  |  |  |  |  |  |  |
| *Moderate to late* | | 202207 | 185335 | 6322 | 1.20 (1.16 to 1.23) | 66 | 2.01 (1.54 to 2.61) | 338 | 2.05 (1.83 to 2.31) |  |  |  |  |  |  |  |  |
| *Very* | | 21091 | 19414 | 1146 | 2.08 (1.93 to 2.24) | 16 | 4.51 (2.73 to 7.47) | 83 | 4.69 (3.76 to 5.85) |  |  |  |  |  |  |  |  |
| *Extremely* | | 5427 | 4990 | 524 | 3.63 (3.22 to 4.09) | 10 | 11.06 (5.88 to 20.80) | 52 | 11.54 (8.74 to 15.22) |  |  |  |  |  |  |  |  |
| **2 years** | | | | | | | | | |  |  |  |  |  |  |  |  |
| **Term** | | 2116767 | 1919681 | 31069 | Ref | 129 | Ref | 947 | Ref |  |  |  |  |  |  |  |  |
| **Preterm** | | 190217 | 172584 | 3518 | 1.26 (1.21 to 1.32) | 36 | 3.12 (2.14 to 4.55) | 155 | 1.74 (1.46 to 2.06) |  |  |  |  |  |  |  |  |
| *Moderate to late* | | 167962 | 152191 | 2848 | 1.16 (1.11 to 1.21) | 28 | 2.75 (1.81 to 4.16) | 114 | 1.45 (1.19 to 1.77) |  |  |  |  |  |  |  |  |
| *Very* | | 17706 | 16222 | 474 | 1.81 (1.62 to 2.03) | 5 | N/A | 31 | 3.66 (2.56 to 5.25) |  |  |  |  |  |  |  |  |
| *Extremely* | | 4549 | 4171 | 196 | 2.87 (2.39 to 3.45) | <5 | N/A | 10 | 4.56 (2.45 to 8.52) |  |  |  |  |  |  |  |  |
| **3 years** | | | | | | | | | |  |  |  |  |  |  |  |  |
| **Term** | | 1708772 | 1469740 | 16621 | Ref | 75 | Ref | 562 | Ref |  |  |  |  |  |  |  |  |
| **Preterm** | | 153254 | 132408 | 1795 | 1.21 (1.14 to 1.28) | 9 | N/A | 100 | 1.79 (1.44 to 2.22) |  |  |  |  |  |  |  |  |
| *Moderate to late* | | 134932 | 116568 | 1493 | 1.14 (1.08 to 1.22) | 7 | N/A | 76 | 1.55 (1.21 to 1.97) |  |  |  |  |  |  |  |  |
| *Very* | | 14565 | 12591 | 214 | 1.52 (1.28 to 1.81) | <5 | N/A | 17 | 3.12 (1.92 to 5.06) |  |  |  |  |  |  |  |  |
| *Extremely* | | 3757 | 3249 | 88 | 2.38 (1.83 to 3.10) | <5 | N/A | 7 | N/A |  |  |  |  |  |  |  |  |
| **4 years** | | | | | | | | | |  |  |  |  |  |  |  | N/A |
| **Term** | | 1219541 | 986481 | 8706 | Ref | 20 | Ref | 331 | Ref |  |  |  |  |  |  |  |  |
| **Preterm** | | 110538 | 90165 | 935 | 1.18 (1.10 to 1.28) | 6 | N/A | 58 | 1.78 (1.34 to 2.37) |  |  |  |  |  |  |  |  |
| *Moderate to late* | | 97233 | 79213 | 778 | 1.12 (1.03 to 1.22) | 5 | N/A | 52 | 1.81 (1.35 to 2.44) |  |  |  |  |  |  |  |  |
| *Very* | | 10594 | 8727 | 121 | 1.59 (1.29 to 1.95) | <5 | N/A | <5 | N/A |  |  |  |  |  |  |  |  |
| *Extremely* | | 2711 | 2225 | 36 | 1.82 (1.19 to 2.80) | <5 | N/A | <5 | N/A |  |  |  |  |  |  |  |  |

N/A: To ensure reliable estimates, only periods with at least 10 events in each group were estimated.

# Supplementary Table 4: Mean ratios for the number of respiratory-related hospitalisations and hazard ratios for respiratory-related mortality comparing preterm and term children for specific ICD-10 blocks.

|  | **ICD-10 Block** | **No. hospitalisations** | **MR (95% CI)** | **No. deaths** | **HR (95% CI)** |
| --- | --- | --- | --- | --- | --- |
| Term | J00-J06 (Acute upper respiratory infections) | 14,279 | Ref | 22 |  |
| Preterm |  | 1,698 | 1.26 (1.19 to 1.33) | 6 | N/A |
| Term | J09-J18 (Influenza and Pneumonia) | 164,677 | Ref | 1263 |  |
| Preterm |  | 21,472 | 1.35 (1.32 to 1.37) | 471 | 3.83 (3.43 to 4.27) |
| Term | J20-J22 (Other acute lower respiratory infections) | 60,178 | Ref | 280 |  |
| Preterm |  | 8,430 | 1.40 (1.37 to 1.44) | 113 | 3.99 (3.19 to 5.00) |

[1] Been JV, Sheikh A. Gestational age misclassification and its effect on disease outcomes after preterm birth. Eur J Epidemiol 2013;28:525–6. https://doi.org/10.1007/s10654-013-9804-0.
